# Association of lower urinary tract symptoms and hip fracture in adults aged ≥ 50 years

**DOI:** 10.1371/journal.pone.0246653

**Published:** 2021-03-03

**Authors:** Pin-Sung Liu, Huei-Kai Huang, Dah-Ching Ding

**Affiliations:** 1 Center for Aging and Health, Hualien Tzu Chi Hospital, Buddhist Tzu Chi Medical Foundation, Hualien, Taiwan; 2 Departments of Family Medicine and Medical Research, Hualien Tzu Chi Hospital, Buddhist Tzu Chi Medical Foundation, and Tzu Chi University, Hualien, Taiwan; 3 Department of Obstetrics and Gynecology, Hualien Tzu Chi Hospital, Buddhist Tzu Chi Medical Foundation, and Tzu Chi University, Hualien, Taiwan; 4 Department of Obstetrics and Gynecology, College of Medicine, Tzu Chi University, Hualien, Taiwan; Medical College of Wisconsin, UNITED STATES

## Abstract

**Aim:**

Lower urinary tract symptoms (LUTS) result in morbidities; however, their association with the occurrence of hip fracture is relatively unknown in the context of Asian studies. The purpose of the study was to investigate this link with the hip fracture risk in Taiwanese men and women aged 50 years and above.

**Materials and methods:**

From 2000 through 2012, a population-based retrospective cohort study was conducted; claims data of 18,976 patients diagnosed with LUTS (dysuria, urinary retention, incontinence, and increased urinary frequency and urgency) were retrieved from Taiwan’s National Health Insurance Research Database. The patients were compared with 1:2 age, sex, and index year-matched controls (comparison group, n = 37,952). The incidence and hazard ratios of the hip fracture risk were calculated by the Cox proportional hazard regression models.

**Results:**

The mean age was 66.2 ± 9.7 years, and the proportion of men was 58.1% in both study groups. Fractures occurred in 772 patients and 1,156 control subjects. The corresponding incidences were 7.0 and 5.0/1000 person-years. Compared to the control subjects, the patients with LUTS had an increased hip fracture risk [adjusted hazard ratio (aHR) = 1.29; 95% confidence interval (CI): 1.17–1.42]. LUTS was independently associated with an increased hip fracture risk in both men (aHR = 1.24; 95% CI: 1.08–1.42) and women (aHR = 1.34; 95% CI: 1.18–1.53) (*p* for interaction = 0.557). Similarly, the subgroup effect of age on hip fracture risks was not found (p for interaction = 0.665).

**Conclusion:**

The study found LUTS was associated with an increased risk of hip fracture. Large-scale prospective studies in diverse populations are required to investigate causalities.

## Introduction

The incidence of hip fractures increases with the rising aging population worldwide; consequently, society and the health care system experience major challenges because of significant disability, morbidity, and mortality [1–3]. Falls, osteoporosis, increased age, dementia, vision loss, and high body weight contribute to the hip fracture risk [4–8]. The incidence of osteoporosis rises with age; it frequently develops during menopause as bone fragility elevates the risk of, or results in fall-related hip fractures [9–11]. Hip fractures lead to increased hospital costs and possibly to secondary health problems or even death. Therefore, prevention of hip fractures and osteoporosis are imperative public health challenges.

Lower urinary tract symptoms (LUTS) are prevalent in elderly men (through benign prostatic hyperplasia) and women (due to menopause), characterized by incomplete voiding, hesitancy, and diminished stream and storage indications like urgency with incontinence, increased frequency, and nocturia. Significant morbidity [12–14] and a potential increase in the risk of falls may be observed; however, a significant connection is to reported. A systematic review of 15 studies stated an overactive bladder (OAB), nocturia, and anticholinergic medications were associated with an increased risk of falls and fracture; however, this was not concurred by the Osteoporotic Fractures in Men (MrOS) prospective cohort study [15–19]. Sporadic studies in Asian countries have compared this association. Studies in Korea have reported a link between LUTS and the risk of falls, possibly predisposing individuals to hip fractures; additionally, the employment of alpha-blockers for LUTS treatment was connected to an increased risk of hip fracture in elderly women [20, 21].

The present study aimed to investigate the association between LUTS and the risk of hip fracture in the elderly population of Taiwan.

## Materials and methods

### Data sources

The present population-based cohort study retrospectively analyzed data retrieved from Taiwan’s National Health Insurance Research Database. The National Health Insurance (NHI) comprises a single-payer mandatory-enrollment system established in 1995 [22]. It provides a comprehensive outpatient, inpatient, and emergency service coverage to >99% of the population contracted with 97% of hospitals and clinics across Taiwan. The Longitudinal Health Insurance Database (LHID) has been established for research purposes; it includes health records of 1 million individuals, with patient’s demographic data and medical claims randomly sampled in the 2000 registry of NHI beneficiaries. To ensure patient privacy and data security, all identifying information was encrypted by the National Health Research Institute. The Research Ethics Committee of the Hualien Tzu Chi Hospital approved the study (REC No: IRB 107-60C), and the need for informed consent was waived. The research was performed in accordance with relevant guidelines/regulations.

### Study population

**Fig 1:**
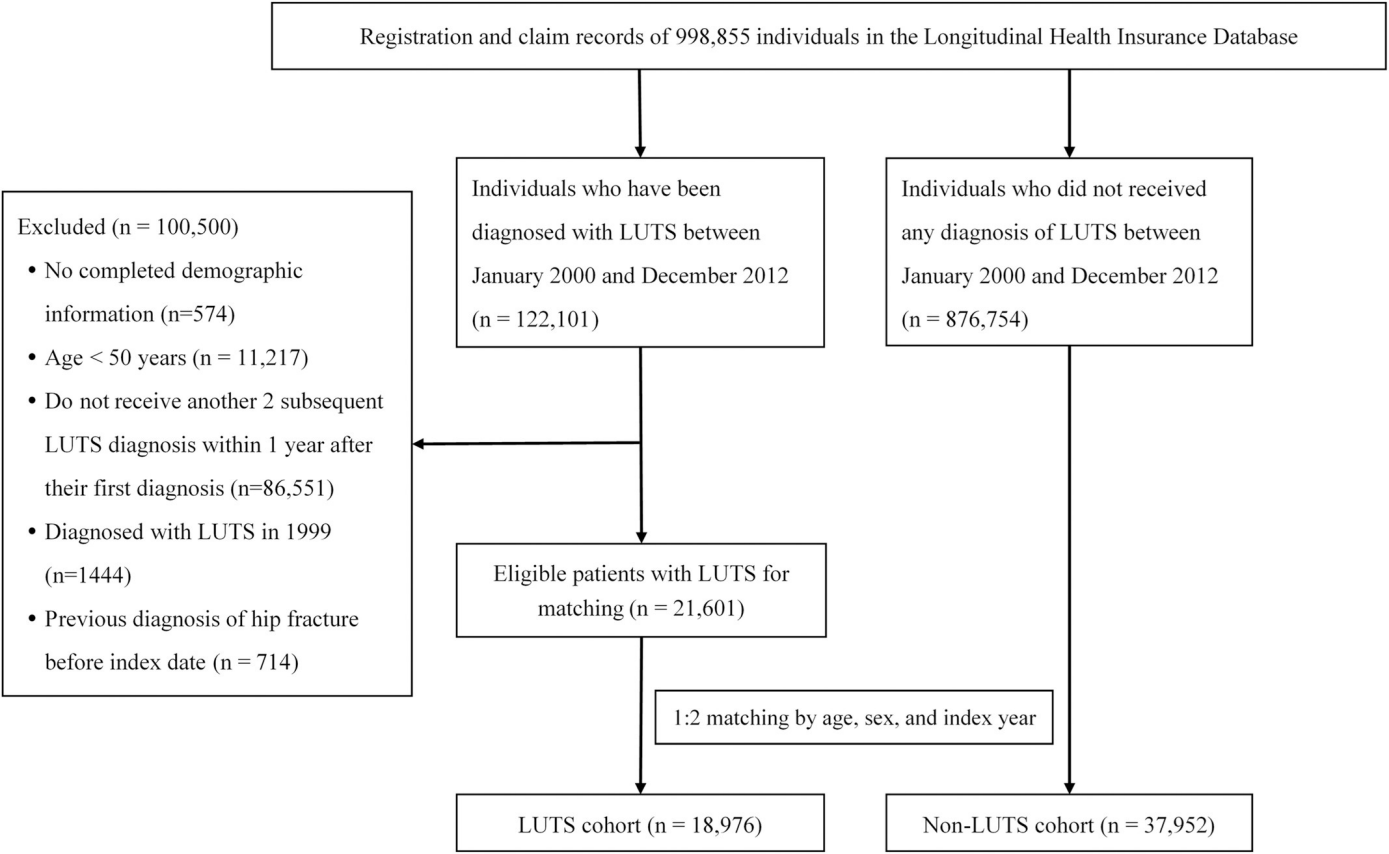
Study flow chart.

The patient selection process is detailed in Fig 1. The study population featured LUTS (exposed) and non-LUTS (comparison) cohorts included in the LHID between 2000 and 2012. Patients aged ≥ 50 years initially diagnosed with LUTS between 2000 and 2012, followed by two subsequent diagnoses within 1 year, were eligible for inclusion. The patients who did not receive 2 subsequent LUTS diagnoses within 1 year of their first diagnosis were excluded. The International Classification of Diseases, Ninth Revision, Clinical Modification (ICD-9-CM) codes 788.1–788.4 and 788.63 were employed to identify patients with LUTS (dysuria, urinary retention, incontinence, and increased urinary frequency and urgency). The third diagnosis was utilized as the index date; follow-up began subsequently. The comparison group comprised a 1:2 age, sex, and index year-matched cohort. The index date was assigned the same as the matched LUTS cases. To ensure the inclusion of newly diagnosed cases, LUTS detected prior to 2000 and hip fracture evaluated before the index date were excluded from both study groups. Patients younger than the specified age limit were ruled out; hip fractures in such cases result from a high-impact trauma event. Additionally, < 2 subsequent diagnoses within 1 year of the initial detection were not considered in the study.

### Outcome measures

The primary outcome was the occurrence of hip fracture (ICD-9-CM codes 820.x and 733.14) during the follow-up. The accuracy of the diagnostic codes had previously been validated [23]. Patients were followed from the index date until the hip fracture, death, withdrawal from the LHID, or the final duration in the database (December 31, 2013). Additionally, the outcome was evaluated following stratification for age and sex.

### Covariates

Baseline patient characteristics considered to be potential confounders were identified by prescription codes, ICD-9-CM codes and procedures of the outpatient and inpatient reimbursement claims; pre-existing comorbidities included were diseases or conditions diagnosed in the year preceding the index date. The baseline medication included drugs prescribed for at least 30 days. The Charlson Comorbidity Index was calculated for each patient [24, 25]. The income was stratified by five levels in New Taiwan Dollars (NTD) as ≥60,000; 40,000−59,999; 20,000−39,999; 15,840−19,999; financially dependent, according to the income-related NHI premiums.

### Statistical analysis

Statistical analysis was performed with Stata (version 13; Stata Corporation, College Station, TX, US). The distinction in the baseline characteristics between the groups was assessed considering the standardized mean difference, with a value < 0.1 considered negligible [26].

The cumulative incidence of the hip fracture was estimated by the Nelson-Aalen method from the initiation of the independent follow-up until the date of event occurrence or censoring. Therefore, the start of the follow-up time for each individual was reset from the calendar date to zero; the end was reset to the actual duration of the observed time. The between-group differences were compared by log-rank tests. The univariable and multivariable Cox proportional regression models were employed to calculate the hazard ratios (HRs) with adjusted values (aHRs), and 95% confidence intervals (CIs) for the hip fracture risk. One multivariable model was adjusted for all covariates listed in Table 1. A second model applied a forward model selection procedure using the score test method with p < 0.1 as the entry-level to include the baseline characteristics for adjustment. Age and sex-stratified sub-analyses were performed, and interaction tests were employed to determine the subgroup effects of age and sex on the hip fracture risk. All covariates listed in Table 1 were adjusted in the multivariable Cox proportional hazards regression model. Two-sided probability values of < 0.05 were considered statistically significant.

**Table 1:** Baseline characteristics of patients with and without LUTS.

	LUTS				SMD
	Yes (n = 18,976)		No (n = 37,952)		
	n	%	n	%	
Age (years)					
50–64	8,451	44.5	16,902	44.5	0.000
65–79	8,769	46.2	17,538	46.2	0.000
≥ 80	1,756	9.3	3,512	9.3	0.000
Mean ± SD	66.2	±9.7	66.2	±9.7	0.000
Sex					
Male	11,033	58.1	22,066	58.1	0.000
Female	7,943	41.9	15,886	41.9	0.000
Income level (NTD)					
Financially dependent	9,674	51.0	18,965	50.0	0.020
15,840–19,999	6,196	32.7	12,981	34.2	0.033
20,000–39,999	1,941	10.2	3,606	9.5	0.024
40,000–59,999	714	3.8	1,478	3.9	0.007
≥60,000	451	2.4	922	2.4	0.003
Comorbidities					
Charlson Comorbidity Index	1.9	±2.1	1.1	±1.7	0.390
Diabetes mellitus	4,026	21.2	5,555	14.6	0.172
Hypertension	8,564	45.1	12,776	33.7	0.236
Thyroid dysfunction	233	1.2	293	0.8	0.046
Depression	894	4.7	697	1.8	0.162
Osteoporosis	1,070	5.6	1,107	2.9	0.135
Medication use					
Steroids	1,250	6.6	1,745	4.6	0.087
Diuretics	2,887	15.2	3,883	10.2	0.150
Statins	2,222	11.7	3,387	8.9	0.092
PPIs	1,094	5.8	1,025	2.7	0.153
Thyroxine	185	1.0	263	0.7	0.031
Antithyroid drugs	86	0.5	121	0.3	0.021
Hypnotics and sedatives	2,874	15.2	2,891	7.6	0.239
Antiosteoporotic drugs	290	1.5	360	1.0	0.052

Continuous variables are reported as means ± standard deviation; categorical variables are reported as numbers and percentages.

Abbreviations: LUTS, lower urinary tract symptoms; NTD, New Taiwan Dollars; PPI, proton pump inhibitor; SMD, standardized mean difference; SD, standard deviation.

### Sensitivity analysis

Considering certain covariates were disproportionate between the groups, a sensitivity analysis was performed with 1:1 propensity score matching; this was based on the previous constructed study population (by 1:2 age, sex, and index year matching) to balance the baseline differences and evaluate the robustness of the study findings. The propensity score was calculated to estimate the probability of obtaining LUTS diagnosis using logistic regression models based on all covariates listed in Table 1. The analysis was performed using the nearest-neighbor matching algorithm without replacement, employing a caliper width equal to 0.2 times the standard deviation of the logit of the propensity score.

## Results

### Patient characteristics

A total of 56,928 patients was enrolled. The LUTS cohort comprised 18,976 subjects; the comparison cohort contained 37,952 patients (Fig 1). Being matched for age, sex, and index year, the proportion of study subjects with comorbidities and specific medication was greater in LUTS than the comparison group. The patient characteristics are displayed in Table 1. The mean follow-up duration was observed as 5.8 years.

### Risk of hip fracture

**Fig 2:**
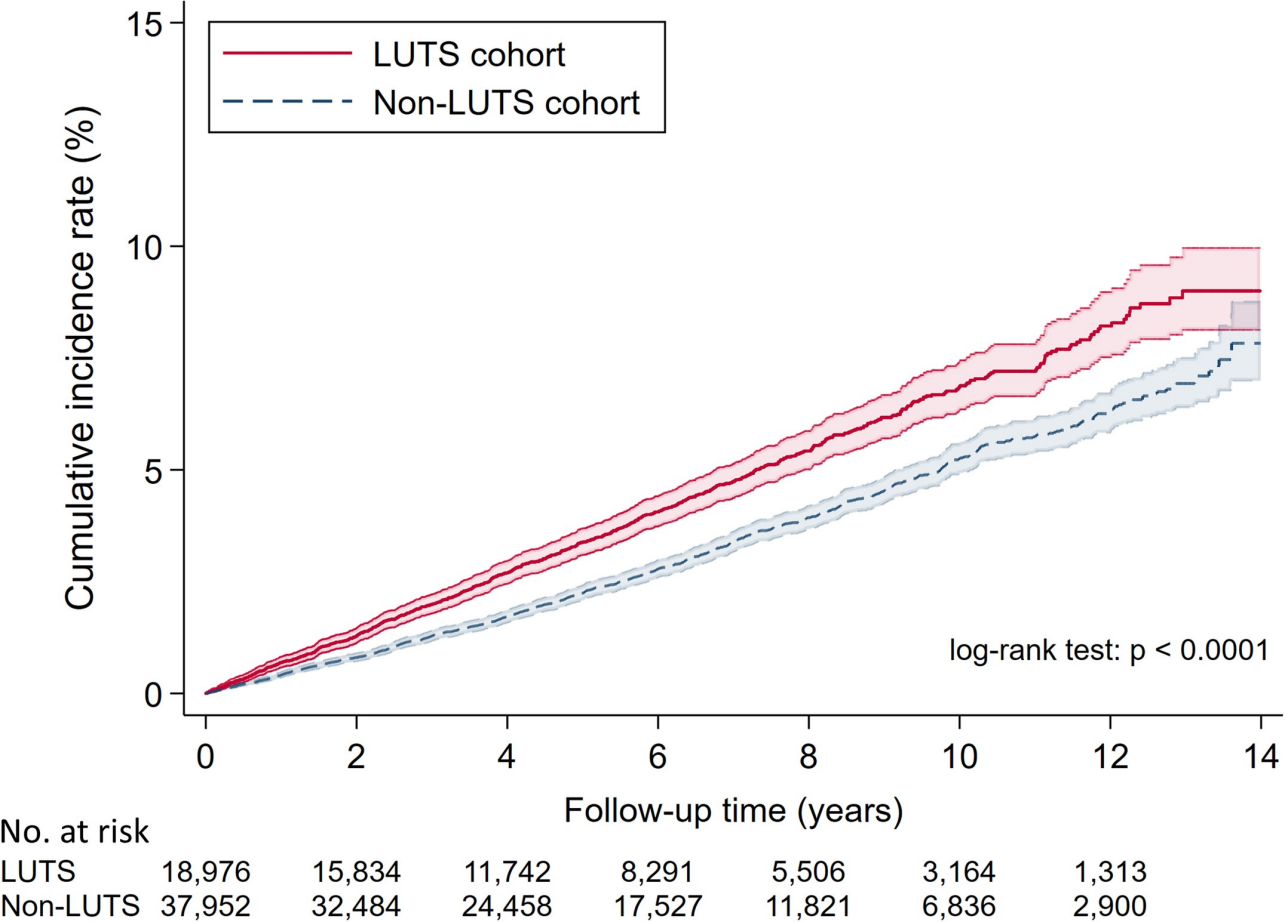
Cumulative incidence curves with 95% confidence intervals (colored zone) of hip fracture in LUTS patients and the comparison group.

During follow-up, 893 and 653 hip fractures were reported in the LUTS and comparison groups, respectively. The Nelson–Aalen estimates in Fig 2 indicated the cumulative risk of hip fracture was greater in the LUTS group (log-rank test, *p* < 0.001). The incidence of hip fracture was 7.0/1000 and 5.0/1000 person-years in the LUTS and comparison groups, respectively. Univariable and multivariable Cox regression analysis findings are provided in Table 2. Based on univariable analysis, LUTS was significantly associated with an increased hip fracture risk (HR = 1.39, 95% CI: 1.27–1.53, p < 0.001); the connection remained significant in both multivariable analysis models (model 1: aHR = 1.29, 95% CI: 1.17–1.42, p < 0.001; model 2: aHR = 1.30, 95% CI: 1.18–1.43, p < 0.001). Table 3 shows the HR of each covariate for the hip fracture risk in the three Cox regression prototypes and those included in the multivariable model 2.

**Table 2:** Risk of hip fracture in patients with and without LUTS.

	LUTS	
	Yes	No
Number of patients	18,976	37,952
Hip fracture events	772	1,156
Person-years	110,737	230,368
Incidence rate*	7.0	5.0
Univariable model		
Crude HR (95% CI)	1.39 (1.27–1.53)	1.00 (ref.)
P value	<0.001	
Multivariable model 1^†^		
Adjusted HR (95% CI)	1.29 (1.17–1.42)	1.00 (ref.)
P value	<0.001	
Multivariable model 2^‡^		
Adjusted HR (95% CI)	1.30 (1.18–1.43)	1.00 (ref.)
*p* value	<0.001	

*Per 1000 person-years

^†^Multivariable Cox regression model adjusted for all covariates shown in Table 1.

^‡^Multivariable Cox regression model that applied a forward selection procedure using score test method with p<0.1 as entry-level to include baseline characteristics for adjustment.

Abbreviations: LUTS, lower urinary tract symptoms; HR, hazard ratio; CI, confidence interval; ref., reference.

**Table 3:** The hazard ratios and 95% confidence intervals of hip fracture event for all of the variables in the three different Cox regression models.

	Univariable model			Multivariable model 1^†^			Multivariable model 2^‡^		
	Crude HR	95% CI	p value	aHR	95% CI	p value	aHR	95% CI	p value
LUTS	1.39	1.27–1.53	<0.001	1.29	1.17–1.42	<0.001	1.30	1.18–1.43	<0.001
Age, year	1.11	1.10–1.11	<0.001	1.10	1.10–1.11	<0.001	1.10	1.10–1.11	<0.001
Sex									
Male	1.00	ref.		1.00	ref.		1.00	ref.	
Female	1.46	1.34–1.60	<0.001	1.60	1.46–1.76	<0.001	1.63	1.48–1.78	<0.001
Income level (NTD)									
Financially dependent	1.00	ref.		1.00	ref.		1.00	ref.	
15,840–19,999	0.80	0.73–0.88	<0.001	1.02	0.93–1.12	0.680	1.02	0.92–1.12	0.722
20,000–39,999	0.25	0.19–0.32	<0.001	0.84	0.64–1.10	0.205	0.83	0.64–1.09	0.191
40,000–59,999	0.15	0.09–0.25	<0.001	0.54	0.32–0.90	0.017	0.53	0.32–0.89	0.016
60,000 above	0.08	0.04–0.20	<0.001	0.33	0.14–0.80	0.014	0.33	0.14–0.80	0.014
Comorbidities									
Charlson comorbidity index	1.20	1.18–1.22	<0.001	1.07	1.04–1.10	<0.001	1.08	1.05–1.11	<0.001
Diabetes mellitus	1.83	1.64–2.03	<0.001	1.27	1.12–1.45	0.000	1.27	1.12–1.45	0.000
Hypertension	1.86	1.70–2.03	<0.001	1.06	0.96–1.17	0.255	-	-	-
Thyroid dysfunction	0.94	0.58–1.54	0.814	0.76	0.40–1.46	0.417	-	-	-
Depression	1.45	1.14–1.83	0.002	1.13	0.88–1.44	0.349	-	-	-
Osteoporosis	2.00	1.70–2.35	<0.001	1.16	0.96–1.40	0.116	-	-	-
Medication use									
Steroids	1.41	1.18–1.70	0.000	1.02	0.85–1.23	0.818	-	-	-
Diuretics	1.89	1.68–2.13	<0.001	1.05	0.92–1.19	0.489	-	-	-
Statins	1.13	0.96–1.32	0.136	0.82	0.70–0.97	0.020	0.83	0.71–0.98	0.029
PPIs	1.28	1.01–1.64	0.043	0.94	0.73–1.20	0.606	-	-	-
Thyroxine	0.99	0.59–1.68	0.974	1.01	0.56–1.82	0.970	-	-	-
Antithyroid drugs	1.28	0.64–2.57	0.482	1.60	0.69–3.68	0.270	-	-	-
Hypnotics and sedatives	1.78	1.57–2.01	<0.001	1.20	1.05–1.38	0.007	1.24	1.09–1.41	0.001
Antiosteoporotic drugs	3.44	2.63–4.50	<0.001	1.31	0.97–1.77	0.079	1.46	1.11–1.92	0.006

^†^Multivariable Cox regression model with adjustment for all covariates listed in Table 1.

^‡^Multivariable Cox regression model that applied a forward model selection procedure using score test method with p < 0.1 as entry-level to include baseline characteristics for adjustment.

Abbreviations: LUTS, lower urinary tract symptoms; HR, hazard ratio; aHR, adjusted hazard ratio; CI, confidence interval; NTD, New Taiwan dollar.

### Risk of hip fracture after stratification by sex and age

Univariable and multivariable regression analysis findings after stratification by sex and age are shown in Table 4. After stratification for sex, LUTS was significantly associated with an increased hip fracture risk in both men (aHR = 1.24, 95% CI: 1.08–1.42; *p* = 0.003) and women (aHR = 1.34, 95% CI: 1.18–1.53; *p* < 0.001) in multivariable model 1. LUTS increased hip fracture risk in patients aged 65–79 years (aHR = 1.29, 95% CI: 1.14–1.45; *p* < 0.001) and ≥80 years (aHR = 1.30, 95% CI: 1.07–1.58; *p* = 0.008). The association between LUTS and increased hip fracture risk in patients aged 50–64 years was not significant (aHR = 1.11, 95% CI:0.87–1.43; *p* = 0.397). The interaction tests did not find any subgroup effects of sex and age on hip fracture risk (sex, *p* for interaction = 0.557; age, *p* for interaction = 0.665).

**Table 4:** Risk of hip fracture in patients with and without LUTS after stratification by sex and age.

Subgroup	Univariable model		Multivariable model 1^†^			Multivariable model 2^‡^		
	Crude HR (95% CI)	*p* value	aHR (95% CI)	*p* value	*p* for interaction	aHR (95% CI)	*p* value	*p* for interaction
Sex					0.557			0.501
Male	1.36 (1.18–1.55)	<0.001	1.24 (1.08–1.42)	0.003		1.26 (1.10–1.45)	0.001	
Female	1.43 (1.26–1.62)	<0.001	1.34 (1.18–1.53)	<0.001		1.35 (1.18–1.53)	<0.001	
Age (years)					0.665			0.851
50–64	1.36 (1.07–1.73)	0.011	1.11 (0.87–1.43)	0.397		1.15 (0.90–1.47)	0.282	
65–79	1.46 (1.30–1.64)	<0.001	1.29 (1.14–1.45)	<0.001		1.29 (1.14–1.45)	<0.001	
≥80	1.37 (1.14–1.65)	0.001	1.30 (1.07–1.58)	0.008		1.34 (1.11–1.62)	0.003	

Patients without LUTS were the reference group in the Cox proportional hazards regression models.

^†^Multivariable Cox regression model adjusted for the covariates in Table 1.

^‡^Multivariable Cox regression model that applied a forward selection procedure using score test method with p < 0.1 as entry-level to include baseline characteristics for adjustment.

Abbreviations: LUTS, lower urinary tract symptoms; HR, hazard ratio; aHR, adjusted hazard ratio; CI, confidence interval.

### Results of sensitivity analysis

In the sensitivity analysis with propensity score matching, the patient characteristics were balanced-well with all standardized mean differences < 0.1; this indicated negligible difference between the groups (S1 Table). After propensity score matching, an elevated risk of hip fracture was observed in patients with LUTS. The aHRs in the multivariable models 1 and 2 were 1.19 (95% CI: 1.07–1.33, p = 0.001) and 1.20 (95% CI: 1.08–1.33, p < 0.001), respectively. This indicated the robustness of the study findings (S2 Table).

## Discussion

An association was observed between LUTS and the hip fracture risk which remained after stratification by sex and age. Compared to the subjects in the comparison group, LUTS patients bore a 29% elevated risk of developing fractures.

Previous studies evaluating the association between LUTS and the hip fracture risk in men have reported inconsistent findings [15, 27–30]. In women, a direct link has been reported [31–33]; however, Schluter et al. failed to demonstrate such an association [27]. In the present study, LUTS was associated with the hip fracture risk in both Taiwanese men and women.

A systematic review did not recognize any association between OAB medications and hip fractures [16]; however, a recent study in Korea reported an increase in the fracture risk (HR 2.19 95% CI: 1.74–2.77) with the use of α-blockers by female patients with LUTS [20]. Another investigation reported male patients consuming prostate-specific α-antagonists faced elevated risks of falls and fractures due to induced hypotension, and a third study reported both medications and nocturia increased the hip fracture risk in elderly individuals [29, 34]. In the present study, the association between LUTS and the increased hip fracture risk was unaffected by sex or age; however, the relationship between OAB medication and the risk of falls or fractures is required to be explored.

It is unknown how LUTS impacts hip fractures. There are several possible explanations. In elderly men, moderate or severe LUTS at night may be associated with an increased risk of falls and hip fractures [30, 35]. Moreover, orthostatic hypotension associated with prostate-specific α-antagonists may lead to falls [34]. Hip fractures may occur in elderly patients with both LUTS and osteoporosis. This is independently associated with an increased risk of fracture and the formation of bladder stones [36–38]. Secondary studies are required to investigate the causal relationships of LUTS with hip fractures.

### Strengths and limitations

The present study data was extracted from insurance claims covering more than 23 million people in Taiwan. However, the long-term follow-up of adults with LUTS experiencing hip fractures was impossible, therefore, a retrospective cohort study including population data was appropriate. The routine long-term clinical follow-up of patients with LUTS allows for identifying those with ICD-9-CM codes for hip fracture in the insurance database. The large population cohort featuring patients with both LUTS and hip fractures was considered to be representative of adults aged ≥50 years in Taiwan. Likewise, the medical practice patterns would be characteristic of the work-up, diagnosis, and treatment.

The limitations feature the inclusion of the LUTS cohort with comorbidities (diabetes, dementia, and medication use involving hypnotics and sedatives) associated with hip fractures [6, 39, 40]; the adjustment for covariates decreased the influence of comorbidities and medication in the present study. Data related to lifestyle habits (smoking and alcohol consumption), substance abuse, chronic hyperglycemia related to hip fracture, body mass index, physical characteristics, and a history of frailty and falls (potentially related to LUTS) were unavailable [41, 42]. Likewise, the unavailability of bone mineral density data occurred due to the non-retrieval of imaging or detailed medical records. This may cause bias, therefore, the present study findings should be interpreted cautiously.

Dual‐energy X‐ray absorptiometry may have been effective for indicating the etiology of fractures; body mass index (BMI) influences bone mineral density, impacting the risk of fracture [43, 44]. Database anonymity made it impossible to contact patients to confirm diagnoses and outcomes; upcoding may result in issues during analysis [45]. Contact with patients could have helped confirm the clinical status, diagnostic information, hospital admission, surgery, and database records, as well as, improve the accuracy of the study. Insurance data may have resulted in selection bias as greater claims ensue increased chances of LUTS diagnosis. Conversely, in another study, patients had under-reported symptoms to their caregivers, resulting in decreased diagnosis [46]. Lastly, the retrospective design using LHID may have introduced an imbalance of health status between the LUTS and comparison groups; selection bias resulted due to differences not evident from the claims data. The ICD-9-CM coding system may not precisely identify the patients in each group. To avoid miscoding, three successive diagnoses of LUTS were required for eligibility. The database was large, but not ethnically diverse; it may apply only to Taiwan. Population-based geographically and ethnically diverse studies would strengthen the findings.

## Conclusions

The study detected an association between LUTS and an increased risk of developing hip fractures. Extensive prospective studies are required to explore whether the association is causative.

## Supporting information

S1 Table Baseline characteristics of patients with and without LUTS after propensity score matching. (PDF)

S2 Table Risk of hip fracture in patients with and without LUTS after propensity score matching. (PDF)

## Abbreviations

aHR: adjusted hazard ratio
CI: confidence interval
HR: hazard ratio
ICD-9-CM: International Classification of Diseases, Ninth Revision, Clinical Modification
LHID: Longitudinal Health Insurance Database
LUTS: lower urinary tract symptoms
MrOS: Osteoporotic Fractures in Men
NHI: National Health Insurance
NTD: New Taiwan Dollars
OAB: overactive bladder
OR: odds ratio

## References

[1] Leibson CL, Tosteson ANA, Gabriel SE, Ransom JE, Melton LJ III. Mortality, Disability, and Nursing Home Use for Persons with and without Hip Fracture: A Population‐Based Study. J Am Geriatr Soc. 2002;50: 1644–1650. 10.1046/j.1532-5415.2002.50455.x . 12366617

[2] Peeters CMM, Visser E, Van de Ree CLP, Gosens T, Den Oudsten BL, De Vries J. Quality of life after hip fracture in the elderly: A systematic literature review. Injury. 2016;47: 1369–1382. 10.1016/j.injury.2016.04.018 . 27178770

[3] Bergström U, Jonsson H, Gustafson Y, Pettersson U, Stenlund H, Svensson O. The hip fracture incidence curve is shifting to the right. Acta Orthop. 2009;80: 520–524. 10.3109/17453670903278282 . 19916682 PMC2823331

[4] Saftari LN, Kwon OS. Ageing vision and falls: a review. J Physiol Anthropol. 2018;37: 11. 10.1186/s40101-018-0170-1 . 29685171 PMC5913798

[5] Huang HK, Lin SM, Loh CH, Wang JH, Liang CC. Association Between Cataract and Risks of Osteoporosis and Fracture: A Nationwide Cohort Study. J Am Geriatr Soc. 2019;Volume 67: 254–260. 10.1111/jgs.15626 . 30281143

[6] Mitchell R, Draper B, Brodaty H, Close J, Ting HP, Lystad R, et al. An 11-year review of hip fracture hospitalisations, health outcomes, and predictors of access to in-hospital rehabilitation for adults ≥ 65 years living with and without dementia: a population-based cohort study. Osteoporos Int. 2020;Volume 31: 465–474. 10.1007/s00198-019-05260-8 . 31897545

[7] Cauley JA, Cawthon PM, Peters KE, Cummings SR, Ensrud KE, Bauer DC, et al. Risk Factors for Hip Fracture in Older Men: The Osteoporotic Fractures in Men Study (MrOS). J Bone Miner Res. 2016;31: 1810–1819. 10.1002/jbmr.2836 . 26988112 PMC5240502

[8] Nasiri Sarvi M, Luo Y. Sideways fall-induced impact force and its effect on hip fracture risk: a review. Osteoporos Int. 2017;28: 2759–2780. 10.1007/s00198-017-4138-5 . 28730547

[9] Cosman F, de Beur SJ, LeBoff MS, Lewiecki EM, Tanner B, Randall S, et al. Clinician’s Guide to Prevention and Treatment of Osteoporosis. Osteoporos Int. 2014;25: 2359–2381. 10.1007/s00198-014-2794-2 . 25182228 PMC4176573

[10] Sözen T, Özışık L, Başaran NÇ. An overview and management of osteoporosis. Eur J Rheumatol Inflam. 2017;4: 46–56. 10.5152/eurjrheum.2016.048 . 28293453 PMC5335887

[11] Tinetti ME, Kumar C. The patient who falls: “It’s always a trade-off”. JAMA. 2010;303: 258–266. 10.1001/jama.2009.2024 . 20085954 PMC3740370

[12] Abrams P, Cardozo L, Fall M, Griffiths D, Rosier P, Ulmsten U, et al. The standardisation of terminology of lower urinary tract function: report from the Standardisation Sub-committee of the International Continence Society. Neurourol Urodyn. 2002;21: 167–178. 10.1002/nau.10052 . 11857671

[13] Litman HJ, Steers WD, Wei JT, Kupelian V, Link CL, McKinlay JB, et al. Relationship of lifestyle and clinical factors to lower urinary tract symptoms: results from Boston Area Community Health survey. Urology. 2007;70: 916–921. 10.1016/j.urology.2007.06.1117 . 17919693 PMC2194647

[14] Kwon H, Kang HC, Lee JH. Relationship between predictors of the risk of clinical progression of benign prostatic hyperplasia and metabolic syndrome in men with moderate to severe lower urinary tract symptoms. Urology. 2013;81: 1325–1329. 10.1016/j.urology.2013.01.042 . 23602796

[15] Marshall LM, Lapidus JA, Wiedrick J, Barrett-Connor E, Bauer DC, Orwoll ES, et al. Lower Urinary Tract Symptoms and Risk of Nonspine Fractures among Older Community Dwelling U.S. Men. J Urol. 2016;196: 166–172. 10.1016/j.juro.2016.02.081 . 26905017 PMC4914455

[16] Szabo SM, Gooch KL, Walker DR, Johnston KM, Wagg AS. The Association Between Overactive Bladder and Falls and Fractures: A Systematic Review. Adv Ther. 2018;35: 1831–1841. 10.1007/s12325-018-0796-8 . 30255417 PMC6223978

[17] Pesonen JS, Vernooij RWM, Cartwright R, Aoki Y, Agarwal A, Mangera A, et al. The Impact of Nocturia on Falls and Fractures: A Systematic Review and Meta-Analysis. J Urol. 2020;203: 674–683. 10.1097/JU.0000000000000459 . 31347956

[18] Suehs BT, Caplan EO, Hayden J, Ng DB, Gaddy RR. The Relationship Between Anticholinergic Exposure and Falls, Fractures, and Mortality in Patients with Overactive Bladder. Drugs Aging. 2019;36: 957–967. 10.1007/s40266-019-00694-5 . 31359329

[19] Szabo SM, Gooch K, Schermer C, Walker D, Lozano-Ortega G, Rogula B, et al. Association between cumulative anticholinergic burden and falls and fractures in patients with overactive bladder: US-based retrospective cohort study. BMJ Open. 2019;9: e026391. 10.1136/bmjopen-2018-026391 . 31061036 PMC6502005

[20] Seo GH, Shim SR, Lee HW, Kim JH, Chun DI, Kim HJ, et al. Risk for Hip Fracture due to Alpha Blocker Treatment in Korean Women: National Health Insurance Database Study. Low Urin Tract Symptoms. 2018;10: 175–180. 10.1111/luts.12157 . 27990752

[21] Hwang TY, Kim SK, Kim KH, Kim JY. Association Between Lower Urinary Tract Symptoms and Falls in Adults Males: Based on the Korean Community Health Survey. Asia Pac J Public Health. 2019;31: 643–651. 10.1177/1010539519878361 . 31561710

[22] Hsing AW, Ioannidis JPA. Nationwide Population Science: Lessons From the Taiwan National Health Insurance Research Database. JAMA Intern Med. 2015;Volume 175: 1527–1529. 10.1001/jamainternmed.2015.3540 . 26192815

[23] Wang WJ, Chao CT, Huang YC, Wang CY, Chang CH, Huang TM, et al. The impact of acute kidney injury with temporary dialysis on the risk of fracture. J Bone Miner Res. 2014;29: 676–684. 10.1002/jbmr.2061 . 23929760

[24] Charlson ME, Pompei P, Ales KL, MacKenzie CR. A new method of classifying prognostic comorbidity in longitudinal studies: development and validation. J Chronic Dis. 1987;40: 373–383. 10.1016/0021-9681(87)90171-8 . 3558716

[25] Elixhauser A, Steiner C, Harris DR, Coffey RM. Comorbidity measures for use with administrative data. Med Care. 1998;36: 8–27. 10.1097/00005650-199801000-00004 . 9431328

[26] Heinze G, Jüni P. An overview of the objectives of and the approaches to propensity score analyses. Eur Heart J. 2011;32: 1704–1708. 10.1093/eurheartj/ehr031 . 21362706

[27] Schluter PJ, Arnold EP, Jamieson HA. Falls and hip fractures associated with urinary incontinence among older men and women with complex needs: A national population study. Neurourol Urodyn. 2018;37: 1336–1343. 10.1002/nau.23442 . 29130513

[28] Asplund R. Hip fractures, nocturia, and nocturnal polyuria in the elderly. Arch Gerontol Geriatr. 2006;43: 319–326. 10.1016/j.archger.2005.12.002 . 16457897

[29] Temml C, Ponholzer A, Gutjahr G, Berger I, Marszalek M, Madersbacher S. Nocturia is an age-independent risk factor for hip-fractures in men. Neurourol Urodyn. 2009;28: 949–952. 10.1002/nau.20712 . 19301408

[30] Hogea B, Bardan R, Sandesc M, Patrascu JMJ, Cumpanas A, Andor B. Are night-time voiding and lower urinary tract symptoms significant risk factors for hip fractures caused by falling during the night in male subjects? Patient Prefer Adherence. 2019;13: 1191–1197. 10.2147/PPA.S205229 . 31413547 PMC6659779

[31] Brown JS, Vittinghoff E, Wyman JF, Stone KL, Nevitt MC, Ensrud KE, et al. Urinary incontinence: does it increase risk for falls and fractures? Study of Osteoporotic Fractures Research Group. J Am Geriatr Soc. 2000;48: 721–725. 10.1111/j.1532-5415.2000.tb04744.x . 10894308

[32] Karabulut A, Simavlı S, Demirtaş Ö, Ök N, Güngör HR, Zümrütbaş A. Evaluation of overactive bladder and nocturia as a risk factor for hip fracture in climacteric women: A matched pair case control study. J Obstet Gynaecol. 2018;38: 252–256. 10.1080/01443615.2017.1349082 . 28903631

[33] Johansson C, Hellström L, Ekelund P, Milsom I. Urinary incontinence: a minor risk factor for hip fractures in elderly women. Maturitas. 1996;25: 21–28. 10.1016/0378-5122(96)01117-6 . 8887305

[34] Welk B, McArthur E, Fraser LA, Hayward J, Dixon S, Hwang YJ, et al. The risk of fall and fracture with the initiation of a prostate-selective α antagonist: a population based cohort study. BMJ. 2015;351: h5398. 10.1136/bmj.h5398 . 26502947 PMC4620650

[35] Parsons JK, Mougey J, Lambert L, Wilt TJ, Fink HA, Garzotto M, et al. Lower urinary tract symptoms increase the risk of falls in older men. BJU Int. 2009;104: 63–68. 10.1111/j.1464-410X.2008.08317.x . 19154508 PMC3031126

[36] Carbone LD, Hovey KM, Andrews CA, Thomas F, Sorensen MD, Crandall CJ, et al. Urinary Tract Stones and Osteoporosis: Findings From the Women’s Health Initiative. J Bone Miner Res. 2015;30: 2096–2102. 10.1002/jbmr.2553 . 25990099 PMC5618440

[37] Lauderdale DS, Thisted RA, Wen M, Favus MJ. Bone mineral density and fracture among prevalent kidney stone cases in the Third National Health and Nutrition Examination Survey. J Bone Miner Res. 2001;16: 1893–1898. 10.1359/jbmr.2001.16.10.1893 . 11585355

[38] Shah D, Badlani G. Treatment of overactive bladder and incontinence in the elderly. Rev Urol. 2002;4 suppl 4: S38–S43. . 16986020 PMC1476020

[39] Donnelly K, Bracchi R, Hewitt J, Routledge PA, Carter B. Benzodiazepines, Z-drugs and the risk of hip fracture: A systematic review and meta-analysis. PLOS ONE. 2017;12: e0174730. 10.1371/journal.pone.0174730 . 28448593 PMC5407557

[40] Hamilton EJ, Davis WA, Bruce DG, Davis TME. Risk and associates of incident hip fracture in type 1 diabetes: The Fremantle Diabetes Study. Diabetes Res Clin Pract. 2017;134: 153–160. 10.1016/j.diabres.2017.10.011 . 29054483

[41] Metcalfe D. The pathophysiology of osteoporotic hip fracture. McGill J Med. 2008;11: 51–57. . 18523524 PMC2322920

[42] Baron JA, Farahmand BY, Weiderpass E, Michaëlsson K, Alberts A, Persson I, et al. Cigarette smoking, alcohol consumption, and risk of hip fracture in women. Arch Intern Med. 2001;161: 983–988. 10.1001/archinte.161.7.983 . 11295961

[43] Gonnelli S, Caffarelli C, Nuti R. Obesity and fracture risk. Clin Cases Miner Bone Metab. 2014;11: 9–14. 10.11138/ccmbm/2014.11.1.009 . 25002873 PMC4064448

[44] Coin A, Sergi G, Benincà P, Lupoli L, Cinti G, Ferrara L, et al. Bone mineral density and body composition in underweight and normal elderly subjects. Osteoporos Int. 2000;11: 1043–1050. 10.1007/s001980070026 . 11256896

[45] Hsieh CY, Su CC, Shao SC, Sung SF, Lin SJ, Kao Yang YH, et al. Taiwan’s National Health Insurance Research Database. Taiwan’s National Health Insurance Research Database: past and future. Clin Epidemiol. 2019;11: 349–358. 10.2147/CLEP.S196293 . 31118821 PMC6509937

[46] Engström G, Walker-Engström ML, Lööf L, Leppert J. Prevalence of three lower urinary tract symptoms in men-a population-based study. Fam Pract. 2003;20: 7–10. 10.1093/fampra/20.1.7 . 12509363

