# Supplementary material for: Association of lower urinary tract symptoms and hip fracture in adults aged ≥ 50 years
Source: PLoS One. 2021 Mar 3;16(3):e0246653. doi: 10.1371/journal.pone.0246653 (PMC7928482; doi:10.1371/journal.pone.0246653)
Supplement: S1 Table — (PDF) [file pone.0246653.s001.pdf]

S1 Table. Baseline characteristics of patients with and without LUTS after propensity score matching

	LUTS				SMD
	Yes (n=18,707)		No (n=18,707)		
	n	%	n	%	
Age (years)					
50–64	8,341	44.6	8,196	43.8	0.016
65–79	8,633	46.2	8,826	47.2	0.021
≥ 80	1,733	9.3	1,685	9.0	0.009
Mean ± SD	66.2	±9.7	66.2	±9.6	0.008
Sex					
Male	10,903	58.3	11,028	59.0	0.014
Female	7,804	41.7	7,679	41.1	0.014
Income level (NTD)					
Financially dependent	9,505	50.8	9,423	50.4	0.009
15,840–19,999	6,124	32.7	6,240	33.4	0.013
20,000–39,999	1,921	10.3	1,910	10.2	0.002
40,000–59,999	709	3.8	707	3.8	0.001
≥60,000	448	2.4	427	2.3	0.007
Comorbidities					
Charlson Comorbidity Index	1.8	±2.1	1.8	±2.0	0.020
Diabetes mellitus	3,904	20.9	4,065	21.7	0.021
Hypertension	8,346	44.6	8,787	47.0	0.047
Thyroid dysfunction	224	1.2	198	1.1	0.013
Depression	793	4.2	667	3.6	0.035
Osteoporosis	1,005	5.4	911	4.9	0.023
Medication use					
Steroids	1,200	6.4	1,135	6.1	0.014
Diuretics	2,790	14.9	2,757	14.7	0.005
Statins	2,163	11.6	2,207	11.8	0.007

PPIs	1,020	5.5	907	4.9	0.027
Thyroxine	179	1.0	177	1.0	0.001
Antithyroid drugs	82	0.4	72	0.4	0.009
Hypnotics and sedatives	2,699	14.4	2,511	13.4	0.029
Antiosteoporotic drugs	274	1.5	252	1.4	0.009

Continuous variables are reported as means ± standard deviation; categorical variables are reported as numbers and percentages.
Abbreviations: LUTS, lower urinary tract symptoms; NTD, New Taiwan Dollars; PPI, proton pump inhibitor; SMD, standardized mean difference
