# Supplementary material for: Association of lower urinary tract symptoms and hip fracture in adults aged ≥ 50 years
Source: PLoS One. 2021 Mar 3;16(3):e0246653. doi: 10.1371/journal.pone.0246653 (PMC7928482; doi:10.1371/journal.pone.0246653)
Supplement: S2 Table — (PDF) [file pone.0246653.s002.pdf]

S2 Table. Risk of hip fracture in patients with and without LUTS after propensity score matching

	LUTS	
	Yes	No
Number of patients	18,707	18,707
Hip fracture events	760	629
Person-years	109,521	109,113
Incidence rate*	6.9	5.8
Univariable model		
Crude HR (95% CI)	1.20 (1.08-1.34)	1.00 (ref.)
p value	<0.001	
Multivariable model 1†		
Adjusted HR (95% CI)	1.19 (1.07-1.33)	1.00 (ref.)
p value	0.001	
Multivariable model 2‡		
Adjusted HR (95% CI)	1.20 (1.08-1.33)	1.00 (ref.)
p value	<0.001	

*Per 1000 person-years

†Multivariable Cox regression model adjusted for all covariates shown in Table 1.

‡Multivariable Cox regression model that applied a forward model selection procedure using score test method with p<0.1 as entry-level to include baseline characteristics for adjustment.

Abbreviations: LUTS, lower urinary tract symptoms; HR, hazard ratio; CI, confidence interval; ref., reference
